# The impact of different alignment strategies on bone cuts for neutral knee phenotypes in total knee arthroplasty

**DOI:** 10.1007/s00167-022-07209-7

**Published:** 2022-11-03

**Authors:** Benjamin L. Schelker, Céline S. Moret, Rüdiger von Eisenhart-Rothe, Heiko Graichen, Markus P. Arnold, Vincent Leclercq, Rolf W. Huegli, Michael T. Hirschmann

**Affiliations:** 1 Department of Orthopedic Surgery and Traumatology, Head Knee Surgery and DKF Head of Research, Kantonsspital Baselland, Bruderholz, 4101 Bottmingen, Switzerland; 2 Department of Clinical Research, Regenerative Medicine & Biomechanics, Research Group Michael T. Hirschmann, University of Basel, 4001 Basel, Switzerland; 3 Department of Orthopedics and Sports Orthopedics, Klinikum Rechts Der Isar, Technical University Munich, Ismaningerstr. 22, 81675 Munich, Germany; 4 Department of Arthroplasty, Sports Medicine and Traumatology, Orthopaedic Hospital Lindenlohe, Indanone 18, 92421 Schwandorf, Germany; 5 LEONARDO, Hirslanden Klinik Birshof, Münchenstein, Switzerland; 6 Symbios, Yverdon-Les-Bains, Switzerland; 7 Institute of Radiology and Nuclear Medicine, Kantonsspital Baselland, Bruderholz, 4101 Bottmingen, Switzerland

**Keywords:** Knee, Arthroplasty, TKA, Alignment, Kinematic, Mechanical, Phenotype, Restricted, Anatomical, Bone cuts

## Abstract

**Purpose:**

The purpose of this study was to simulate and visualise the influence of the alignment strategy on bone resection in neutral knee phenotypes. It was hypothesised that different amounts of bone resection would be required depending on the alignment strategy chosen. The hypothesis was that by visualising the corresponding bone cuts, it would be possible to assess which of the different alignment strategies required the least change to the soft tissues for the chosen phenotype but still ensured acceptable component alignment and could, therefore, be considered the most ideal alignment strategy.

**Methods:**

Simulations of the different alignment strategies (mechanical, anatomical, restricted kinematic and unrestricted kinematic) regarding their bone resections were performed on four common exemplary neutral knee phenotypes. *NEU*_*HKA*_*0° VAR*_*FMA*_* 90° VAL*_*TMA*_*90°, NEU*_*HKA*_*0° NEU*_*FMA*_* 93° NEU*_*TMA*_*87°, NEU*_*HKA*_*0° VAL*_*FMA*_* 96° NEU*_*TMA*_*87° and* NEU_HKA_0° *VAL*_*FMA*_* 99° VAR*_*TMA*_*84°.* The phenotype system used categorises knees based on overall limb alignment (i.e. hip knee angle) but also considers joint line obliquity (i.e. TKA and FMA) and has been used globally since its introduction in 2019. These simulations are based on long leg weightbearing radiographs. It is assumed that a change of 1° in the alignment of the joint line corresponds to correspond to 1 mm of distal condyle offset.

**Results:**

In the most common neutral phenotype *NEU*_*HKA*_*0° NEU*_*FMA*_* 93° NEU*_*TMA*_*87°*, with a prevalence of 30%, bone cuts remain below 4 mm regardless of alignment strategy. The greatest changes in the obliquity of the joint line can be expected for the mechanical alignment of the phenotype *NEU*_*HKA*_*0° VAL*_*FMA*_* 99° VAR*_*TMA*_*84°* where the medial tibia is raised by 6 mm and the lateral femur is shifted distally by 9 mm. In contrast, the *NEU*_*HKA*_*0° VAR*_*FMA*_* 90° VAL*_*TMA*_*90°* phenotype requires no change in joint line obliquity if the mechanical alignment strategy is used.

**Conclusion:**

Illustrations of alignment strategies help the treating surgeon to estimate the postoperative joint line obliquity. When considering the alignment strategy, it seems reasonable to prefer a strategy where the joint line obliquity is changed as little as possible. Although for the most common neutral knee phenotype the choice of alignment strategy seems to be of negligible importance, in general, even for neutral phenotypes, large differences in bone cuts can be observed depending on the choice of alignment strategy.

## Introduction

The optimal alignment strategy for total knee arthroplasty is still under debate [19]. To date, the concept of mechanical alignment (MA) aiming to align the leg neutrally and to load the knee prosthesis as evenly as possible has been the method of choice. This strategy has yielded excellent survival rates, but a significant proportion of patients remained dissatisfied regarding the functional outcomes despite the use of advanced implant designs and improved precision of surgical technique [4, 18]. Newer alignment strategies such as the phenotype alignment, unrestricted (KA) and restricted kinematic alignment (rKA) aim to restore pre-arthritic alignment, which would lead to more natural knee kinematics and, thus, improve the functional outcome [3, 5]. However, some knee surgeons are concerned about the long-term survival, as it is assumed that maintaining certain preoperative varus deformity might lead to implant failure over the years and may require revision surgery [2]. Hirschmann et al. [7, 8] assessed the tibial and femoral joint lines in relation to overall limb alignment in the coronal plane in non-osteoarthritic and osteoarthritic knees. Based on this analysis, the authors identified common coronal knee phenotypes and observed that mechanical, anatomical and restricted kinematic alignment matched phenotypes in only 5%, 20% and 51% of the non-osteoarthritic population, respectively. In fact, recent studies have shown that knee phenotypes undergoing total knee arthroplasty are very heterogeneous and are significantly more in varus or valgus than in a control group without OA [6, 10]. These results support the fact that one alignment strategy does not suit all patients and a more personalised approach should be considered. One can hypothesise that the choice of alignment strategy is more important in varus or valgus knee phenotypes than in neutral phenotypes, as the changes in alignment are likely to be greater, especially when the mechanical and anatomical alignment strategy is applied. This simulation study is limited to the bony configuration of the joint; for simplicity, the different ligament variants of each phenotype are not considered and, hence, remain potential topics for future studies.

The aim of this study was, therefore, to perform a simulation study to visualise (1) how the coronal limb alignment of the most common neutral knee phenotypes is altered by current systematic and personalised alignment strategies and (2) whether these illustrations can be used to establish basic recommendations for selecting the best alignment strategy for those specific phenotypes. It was hypothesised that a patient with a preoperative neutral global limb alignment would have different bone resections depending on the specific knee phenotype and alignment strategy used. However, regardless of the alignment strategy used, the overall alignment of the neutral limb should not be changed in neutral phenotypes.

## Materials and methods

**Fig. 1:**
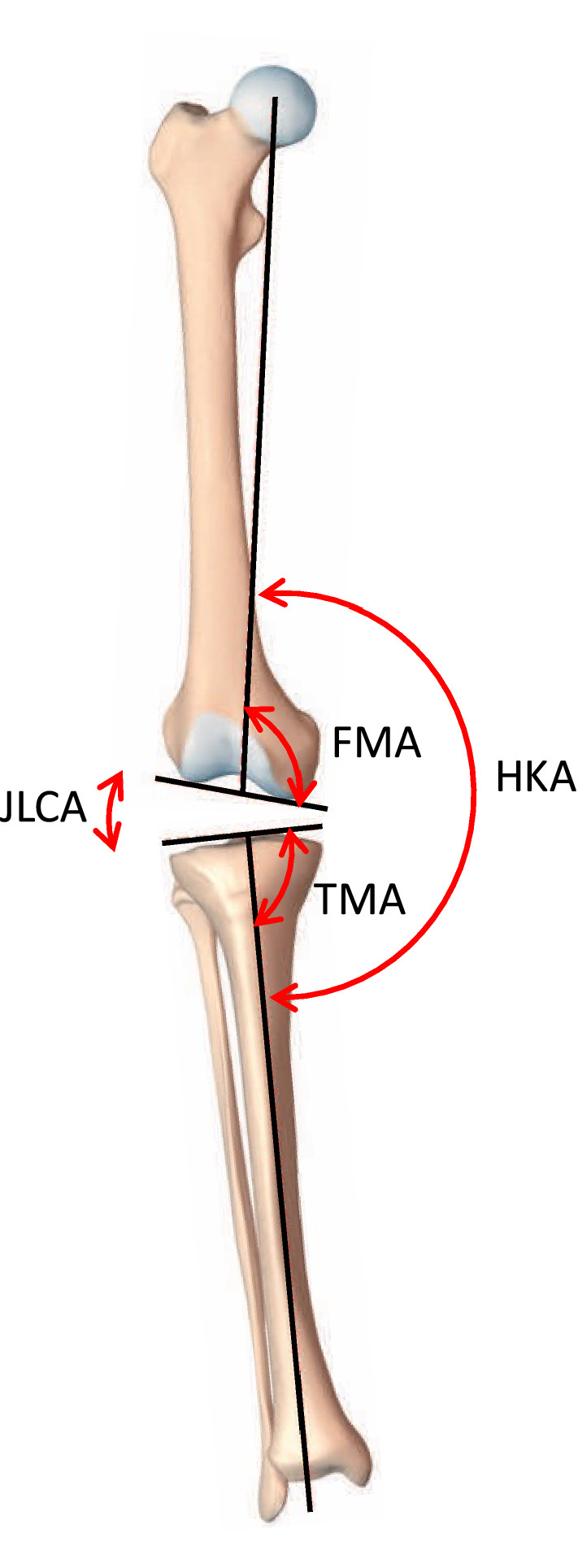
The hip knee ankle angle (*HKA*) is formed by the lines connecting the centre points of the femoral head, the knee and the talus; FMA is the angle between the mechanical axis of the femur and a tangent to the distal femoral condyles; TMA is defined as the angle between the mechanical axis of the tibia and a tangent to the proximal articular surface of the tibia. The joint line convergence angle (JLCA) is the angle between a tangent to the proximal articular surface of the tibia and the tangent of the femoral condyles

The coronal alignment of different functional knee phenotypes is described and assessed in this simulation study with regards to the imbalance of distal bone cuts and the resulting distal femoral joint line changes. The hip–knee–ankle angle (HKA), the distal mechanical femur angle (FMA) and the proximal mechanical tibia angle (TMA) are displayed in Fig. 1. All angles are measured medially. Neutral (NEU) tibial and femoral as well as limb alignments are defined as 93° (± 1.5°) for the FMA, 87° (± 1.5°) for the TMA and 180°(± 1.5°) for HKA. Consequently, a value above 94.5° for FMA and 181.5° for HKA, or above 88.5° for TMA corresponds to a valgus (VAL) alignment and a value below 85.5° for TMA and 178.5° for HKA, or below 91.5° for FMA corresponds to a varus (VAR) alignment. Also shown in the illustrations is the joint line convergence angle (JLCA), which is the angle of intersection of the distal femoral and proximal tibial joint lines. For better illustration, neutral angles are shown in green, varus in blue and valgus in red. A change in 1° in the joint line orientation is considered to correspond to 1 mm of distal condyle offset.

**Fig. 2:**
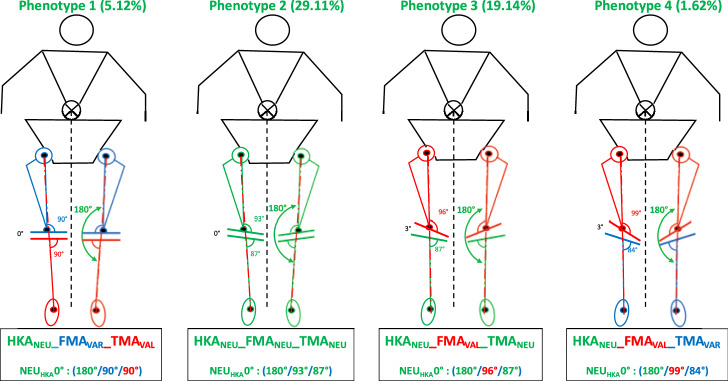
The four common or exemplary neutral (NEU) “native” phenotypes

**Fig. 3:**
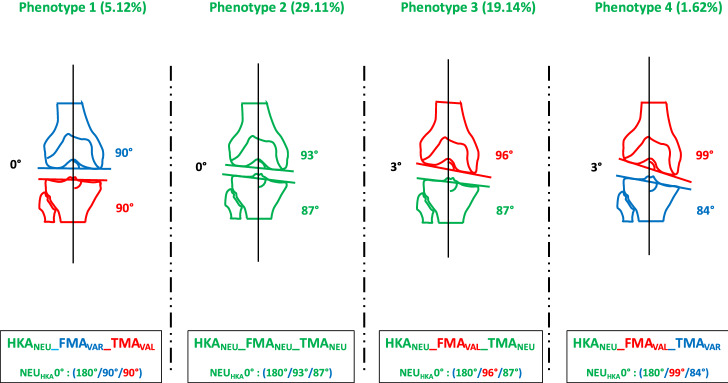
Four common or exemplary neutral (NEU) phenotypes with the different joint line obliquities

As categorising patients only according to the overall alignment of the leg, i.e. dividing them into varus, valgus and neutral patients, does not reflect the variability of coronal alignment, Hirschmann et al. [8] introduced a system to categorise patients according to the alignment of the joint lines of the tibia (TMA) and femur (FMA) in relation to the overall alignment (HKA). The phenotypes are named in the following order. The first abbreviation (NEU, VAR, VAL) indicates the direction of alignment. The second (HKA, FMA and TMA) indicates the measured angle. The last value (0°, 3° and 6°) shows the mean deviation of the phenotype from the mean value of 180° for HKA and covers a range of ± 1.5° from this mean value. The values for FMA and TMA are shown as absolute values and also cover a range of ± 1.5°. For example, the knee phenotype NEU_HKA_0° + NEU_FMA_93° + NEU_TMA_87° means that a patient has a neutral HKA (180° ± 1.5), a neutral FMA (93° ± 1.5) and a neutral TMA (87° ± 1.5). Figures 2 and 3 show four possible phenotypes. The phenotypes chosen for the simulation are a selection of two of the most common and two somewhat rarer but exemplary neutral phenotypes (FMA & TMA can be either NEU, VAL or VAR) to best represent the spectrum of possible neutral phenotypes (Figs. 2, 3). In a population of 2810 subjects with osteoarthritis, examined regarding their knee phenotypes, there are 371 (13.2%) patients with a neutral total limb alignment. From this cohort of 371 patients, the frequencies of the four chosen phenotypes were calculated (Table 1). The first phenotype represents a phenotype that is close to the MA target, the second phenotype is close to an AA target, and the last two phenotypes have greater joint line obliquities. The different simulations are performed for these specific knee phenotypes.

**Table 1:** Basic characteristics of the cohort of osteoarthritic knees

	Overall
Number of patients	371
Age (years), mean (± SD)	70.5 (± 9.5)
Male gender, *n* (%)	130 (35%)

Mechanical alignment (MA) aims to position both the femoral and tibial components perpendicular to the mechanical axis of the corresponding bone to achieve a HKA of 180°. A HKA deviation of ± 3° is considered acceptable (Table 2).

**Table 2:**
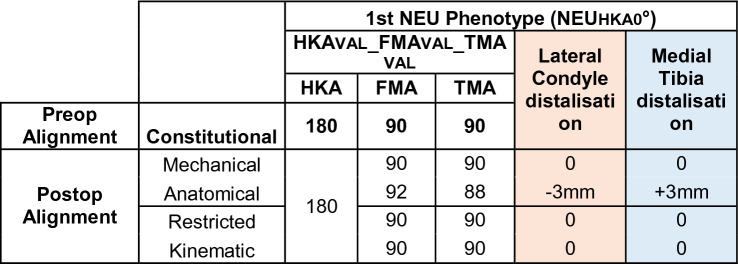
Changes in the medial and lateral distal offset depending on the chosen alignment philosophy

The anatomical alignment (AA) technique has the goal to create an oblique joint line of 2–3° from the perpendicular to the mechanical axis, respectively, of 2–3° of valgus for the femur and 2–3° of varus for the tibia in relation to the mechanical axis [19]. The target value of the HKA is 180°.

The kinematic alignment (KA) technique aims to restore pre-arthritic limb and joint line alignment of TMA, FMA and HKA while sparing the ligamentous structures. The KA implantation is initiated with the femoral cut. The tibial cut is then performed parallel to the femoral joint line. Any ligament balancing is adjusted by tibial bone cuts [15].

The restricted kinematic alignment (rKA) technique aims to restore constitutional joint lines and limb alignment, taking into account a safe zone, i.e. the HKA should remain ± 3° of 180° and the FMA and TMA should be ± 5° in relation to the mechanical axis [23].

The different bone cuts according to the chosen alignment strategy could lead to a change in the joint line obliquity and to a change in the joint line height.

The joint line obliquity is defined as the angle formed by a parallel line to the floor and the joint line [9]. For the change in joint line height, a distinction must be made between a symmetrical and an asymmetrical change in joint line height. There is evidence in the literature that a symmetrical joint line height change might indeed have a negative impact on clinical outcome [22]. However, it is unclear whether and at what threshold asymmetric joint line height shifts may have an impact on clinical outcome (Table 3).

**Table 3:**
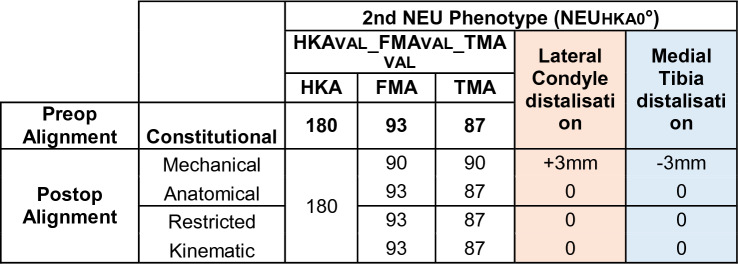
Changes in the medial and lateral distal offset depending on the chosen alignment philosophy

## Results

### Phenotype 1: NEU_HKA_0° VARFMA 90° VAL_TMA_90° (Fig. 4)

**Fig. 4:**
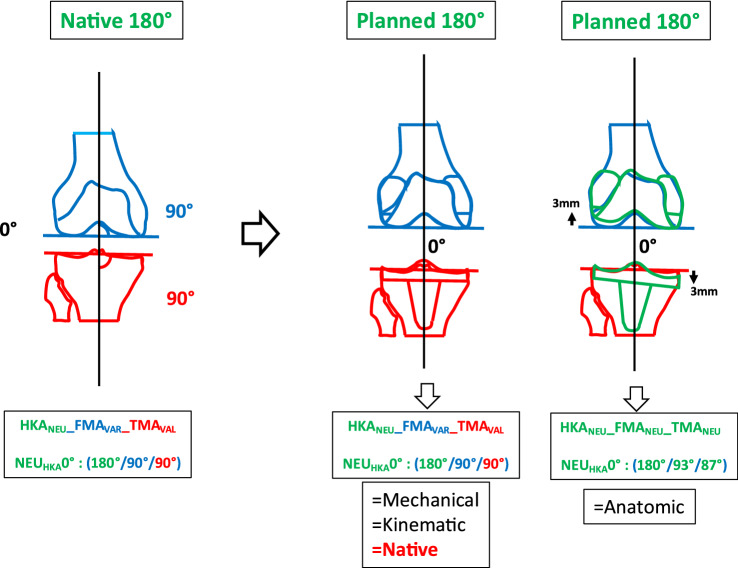
Phenotype 1: NEU_HKA_0° VAR_FMA_ 90° VAL_TMA_90°

The prevalence of this phenotype in the described population of patients with knee OA and neutral phenotype is 5.12%. This phenotype corresponds to the alignment goal of the MA. Therefore, no proximal or distal shift of the medial and/or lateral condyles is required when applying MA, rKA or KA. Assuming that an AA is performed (Table 4), the medial tibial plateau is distalised and the lateral femoral condyle is proximalised by 3 mm.

**Table 4:**
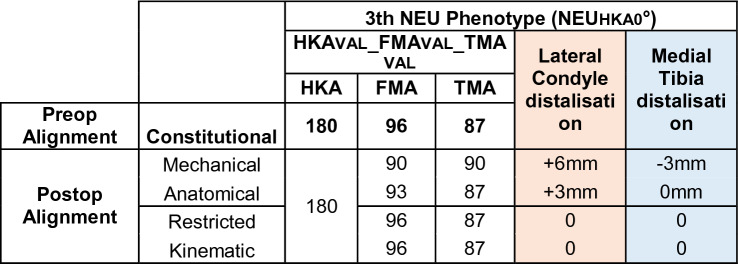
Changes in the medial and lateral distal offset depending on the chosen alignment philosophy

### Phenotype 2: NEU_HKA_0° NEUFMA 93° NEU_TMA_87° (Fig. 5)

**Fig. 5:**
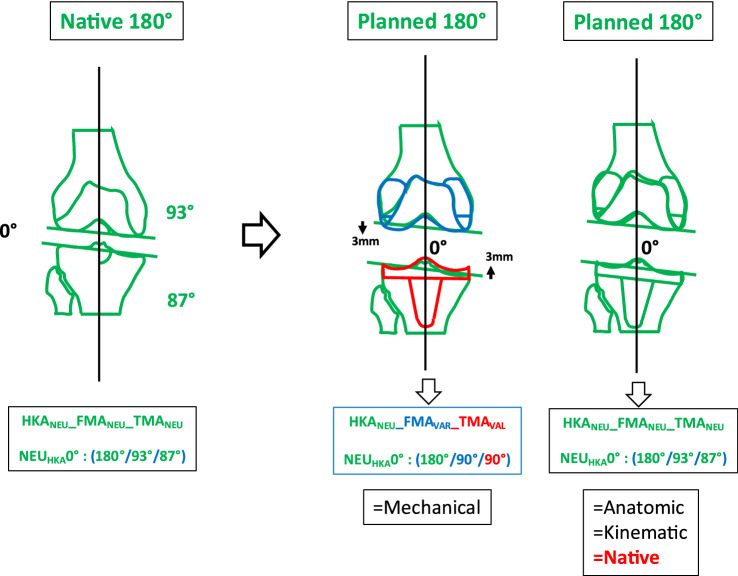
Phenotype 2: NEU_HKA_0° NEU_FMA_ 93° NEU_TMA_87°

The prevalence of this phenotype in the osteoarthritic neutral patient population is 29.11%. This phenotype corresponds to the alignment goal of AA. Therefore, when applying AA or KA, no distalisation of the condyles occurs. When using MA, distalisation of the medial tibial plateau and proximalisation of the lateral femoral condyle by 3 mm occurs.

### Phenotype 3: NEU_HKA_0° VALFMA 96° NEU_TMA_87° (Fig. 6)

**Fig. 6:**
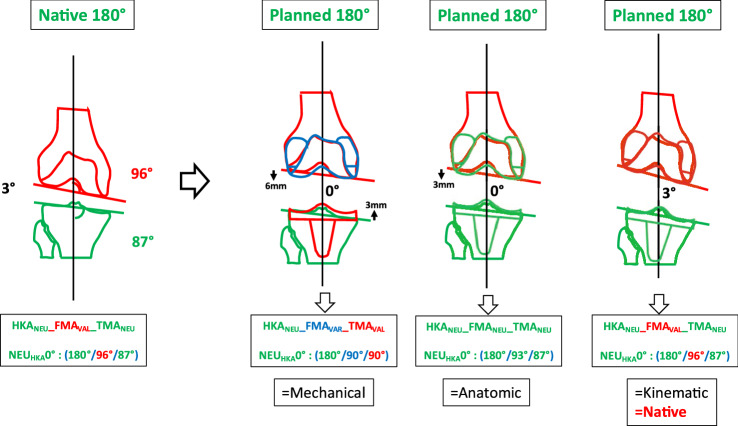
Phenotype 3: NEU_HKA_0° VAL_FMA_ 96° NEU_TMA_87°

Prevalence of this phenotype in the osteoarthritic population corresponds to 19.14%. When using KA no change of the joint line obliquity is required (Table 5). When using MA, elevation of the medial tibial plateau and distalisation of the lateral femoral condyle by 6 and 3 mm, respectively, occurs. The phenotype 3 presents a JLCA of 3°. For the systematic alignment strategies, the JLCA is adjusted; for KA, the JLCA is left unchanged.

**Table 5:**
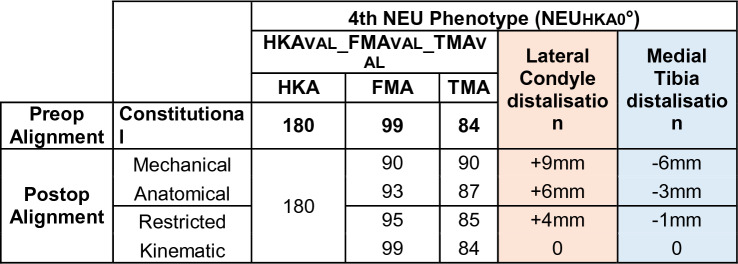
Changes in the medial and lateral distal offset depending on the chosen alignment philosophy

### Phenotype 4: NEU_HKA_0° VALFMA 99° VAR_TMA_84° (Fig. 7)

**Fig. 7:**
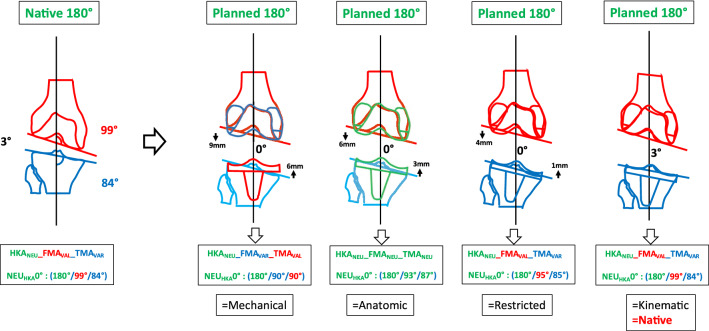
Phenotype 4: NEU_HKA_0° VAL_FMA_ 99° VAR_TMA_84°

The prevalence of this phenotype in the OA population is 1.62%. When using KA, no change of the joint line obliquity is required. The required adjustments of the medial and lateral offsets depending on the alignment strategy are shown in Table 5. Phenotype 4 also has a JLCA of 3°. For MA and AA the JLCA is set to 0° for KA the JLCA remains unchanged.

## Discussion

The main findings of this study were that depending on the alignment chosen, the four either common or exemplary neutral (NEU) knee phenotypes caused either none or partly significant changes in joint line obliquity and offset. In phenotypes 1 and 2, which both represent together over a third of all neutral phenotypes according to the cohort of OA patients assessed, the changes in distal offset were below 4 mm medially and laterally regardless the alignment strategy chosen, which can be considered as an approximate threshold for offset changes with negligible clinical relevance. However, the evidence for this threshold is mostly based on experience and scarce evidence. The only available evidence comes from a systematic review by van Lieshout et al. [22]. Here, the authors showed a correlation between an elevated joint line and a worse clinical outcome. The authors, thus, recommended avoiding an increase in the joint line of more than 4 mm. However, the study considered symmetrical changes in joint line height. In the present study, the changes in joint line height were asymmetrical. Therefore, it is still under debate how much change in the medial and/or lateral joint line heights a knee can tolerate or compensate. Truly personalised alignment concepts such as unrestricted kinematic alignment do not lead to changes in joint line obliquity, but the long-term implant survival of particularly an obliquely aligned femoral component is still not sufficiently investigated. Hence, it needs a safe transition from mechanical to personalised alignment. It is therefore currently a compromise of what the fixation of the TKA can tolerate and what the optimal alignment for the best function is.

In the present study for *phenotype 1 NEU*_*HKA*_*0° VAR*_*FMA*_* 90° VAL*_*TMA*_*90° *(Fig. 4) and *phenotype 2 NEU*_*HKA*_*0° NEU*_*FMA*_* 93° NEU*_*TMA*_*87° *(Fig. 5), whichever alignment strategy is chosen is unlikely to have a relevant effect on clinical outcome because the changes in joint line height are less than 3 mm in each of the compartments. Thus, for *phenotypes 3* *NEU*_*HKA*_*0° VAL*_*FMA*_* 96° NEU*_*TMA*_*87°* and especially *4 NEU*_*HKA*_*0° VAL*_*FMA*_* 99° VAR*_*TMA*_*84°*, the choice of alignment strategy could potentially have an impact on clinical outcome by altering the joint line obliquity, respectively, lead to an asymmetric change of the joint height. The alteration of the joint line alignment may therefore also lead to patellofemoral problems as the elevated joint lines change the direction of the load as well as contact forces at the patella [11, 20]. In general, there seems to be a consensus in the orthopaedic surgeon community that not every anatomy is considered healthy or constitutional, respectively, that certain configurations are biologically inferior and, therefore, should not be reproduced. However, as shown in the simulations, the more extreme joint configurations require greater bone resections for the systematic alignment strategies such as MA or AA and therefore involve a greater risk of a detrimental alteration of the flexion axis, the joint line orientation and a possible adverse change in the knee kinematics. The Classification of Coronal Alignment of the Knee (CPAK) classifies knee phenotypes into 9 different types based on their arithmetic HKA and joint line obliquity and recommends which alignment should be used depending on the CPAK type [16]. The CPAK types II, V and VIII correspond to neutral limb alignment. CPAK type V corresponds to *phenotype 1 NEU*_*HKA*_*0° VAR*_*FMA*_* 90° VAL*_*TMA*_*90°* and CPAK type II corresponds to *phenotype 2 NEU*_*HKA*_*0° NEU*_*FMA*_* 93° NEU*_*TMA*_*87°*. The authors’ conclusion to apply MA to CPAK type V and AA to CPAK type II is consistent with the results of the study presented here, as selected strategies do not require a change in the obliquity of the joint line and are therefore more likely to achieve a balanced soft tissue envelope without the need for ligament release.

How oblique a TKA can be placed without increasing wear is still unclear. Radiostereometric analysis demonstrated that varus alignment of the tibial component, but interestingly not the overall alignment of the limb, causes greater migration of the tibial implant [21]. However, other long-term studies contradict this result and see no connection between coronal alignment and implant survival [1]. Perhaps custom-made implants allow more extreme implant positions without increased risk of loosening and without the problem of patellofemoral joint malalignment that can occur with standard knee implants. However, clear evidence of a benefit is still lacking here as well [12, 17].

The fact that the anticipated improvements in clinical outcome of KA-TKA compared to MA-TKA have not yet been observed or have remained unclear supports the findings from the present simulation study [14]. It appears that the data analysis should be done for neutral, varus and valgus phenotypes separately. Otherwise, possible differences in varus or valgus phenotypes are not found as these are obscured by the fact that NEU phenotypes show no clinically relevant and significant differences. “One stands in the forest, and due to the fog one does not see the trees.” Hence, it is clearly recommended to report patients` outcomes as well as comparisons between different surgical techniques as well as alignment philosophies by NEU, VAR or VAL phenotypes separately. This is in agreement with a study by Luan et al. [13] who found that KA may actually have better outcomes in more severe varus patients.

The present study has some limitations. Only the effects of alignment on four exemplary neutral phenotypes in the coronal plane were investigated. In further steps, however, such simulations could be carried out for many more phenotypes and made more easily accessible with a corresponding software solution. Personalised alignment strategies are under constant development and a mix of different strategies is currently used [15]. This could not be considered in this study. The effects in the sagittal and axial planes were not investigated. The study deals with the alignment of the extended leg. However, the coronal alignment also has a significant influence on the flexion gap geometry. However, this would complicate things further, yet should be investigated in more depth in future studies.

## Conclusion

Visualisations of the alignment strategies help to estimate the postoperative joint line obliquity. In principle, when choosing the alignment strategy, it seems reasonable to prefer a strategy where the joint line alignment is changed as little as possible. For the most common neutral knee phenotype, the choice of alignment strategy does seem to be of negligible importance, so that a variety of different alignment strategies can be chosen. However, for most of the neutral phenotypes, the choice of alignment strategy seems to be of importance, and illustrations like the ones in this study can help in daily clinical work to choose the right alignment strategy.
